# Circadian challenge of astronauts’ unconscious mind adapting to microgravity in space, estimated by heart rate variability

**DOI:** 10.1038/s41598-018-28740-z

**Published:** 2018-07-10

**Authors:** Kuniaki Otsuka, Germaine Cornelissen, Yutaka Kubo, Koichi Shibata, Mitsutoshi Hayashi, Koh Mizuno, Hiroshi Ohshima, Satoshi Furukawa, Chiaki Mukai

**Affiliations:** 1 Executive Medical Center, Totsuka Royal Clinic, Tokyo Women’s Medical University, Tokyo, Japan; 2 Halberg Chronobiology Center, University of Minnesota, Minneapolis, Minnesota USA; 3 Department of Medicine, Tokyo Women’s Medical University, Medical Center East, Tokyo, Japan; 4 Faculty of Education, Tohoku Fukushi University, Miyagi, Japan; 5 Space Biomedical Research Group, Japan Aerospace Exploration Agency, Tokyo, Japan

## Abstract

It is critical that the regulatory system functions well in space’s microgravity. However, the “intrinsic” cardiovascular regulatory system (β), estimated by the fractal scaling of heart rate variability (HRV) (0.0001–0.01 Hz), does not adapt to the space environment during long-duration (6-month) space flights. Neuroimaging studies suggest that the default mode network (DMN) serves a broad adaptive purpose, its topology changing over time in association with different brain states of adaptive behavior. Hypothesizing that HRV varies in concert with changes in brain’s functional connectivity, we analyzed 24-hour HRV records from 8 healthy astronauts (51.8 ± 3.7 years; 6 men) on long (174.5 ± 13.8 days) space missions, obtained before launch, after about 21 (ISS01), 73 (ISS02), and 156 (ISS03) days in space, and after return to Earth. Spectral power in 8 frequency regions reflecting activity in different brain regions was computed by maximal entropy. Improved β (p < 0.05) found in 4 astronauts with a positive activation in the “HRV slow-frequency oscillation” (0.10–0.20 Hz) occurred even in the absence of consciousness. The adaptive response was stronger in the evening and early sleep compared to morning (p = 0.039). Brain functional networks, the DMN in particular, can help adapt to microgravity in space with help from the circadian clock.

## Introduction

As humans venture into space, it is critical that the regulatory system should remain fully functional. Humans, including their cardiovascular system must first acclimate to a new environment in order to survive. Spaceflight dramatically alters cardiovascular dynamics^1–5^. Baroreflex sensitivity fluctuates and blood volume distribution is altered, affecting neural mechanisms involved in dynamic cardiovascular coordination. Cardiac output and stroke volume reportedly increase by 35–41% after 3–6 months in space^4^. Parasympathetic activity is reduced in space^3^, with no major difference in sympathetic nerve activity. Blood pressure is reduced by 8–10 mmHg related to a 39% decrease in systemic vascular resistance, not resulting from sympathetic nervous activity suppression, while the nocturnal dip is maintained. Understanding how organisms – and their neural cardiovascular coordination – adjust in space remains a challenge, as mechanisms are diverse and complex^5–9^.

The “intrinsic” cardiovascular regulatory system, estimated by the fractal scaling of heart rate variability (HRV) (slope “β”), did not adapt to microgravity after 6 months in space^10–12^, whereas the circadian rhythm of heart rate (HR) did improve^10^, an important feature since circadian disruption adversely affects health^13–17^. Beyond circadian rhythms, multi-frequency oscillations^12,18–20^ include the ~90-minute basic rest-activity cycle (BRAC)^12^. Most HRV changes in space apparently relate to the BRAC, which was amplified ~3-fold for specific HRV endpoints^12^, perhaps as a way to adapt to microgravity during long-duration spaceflight.

**Figure 1:**
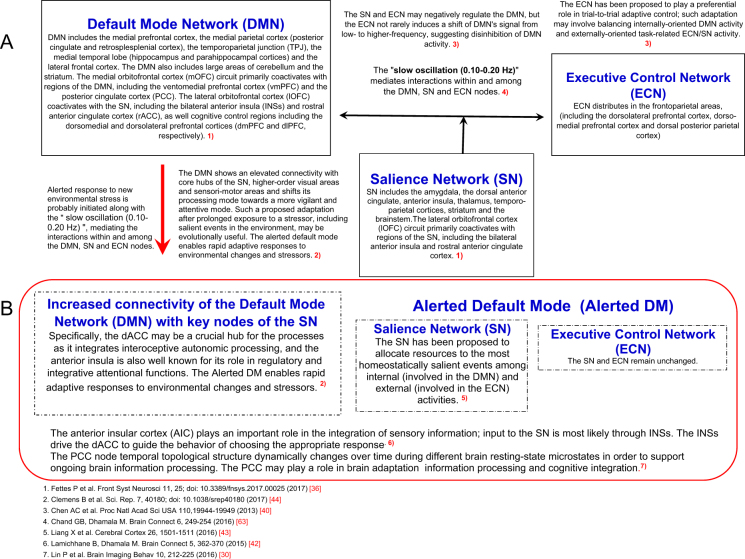
Interactions among the default mode, salience and executive control networks, and shift to an alerted default mode for adaptive responses to environmental changes and stressors. (**A**) Dynamic functional connectivity patterns are tightly linked to adaptive behavior as a guide toward optimal behavioral performance^24^. The DMN is most active during the resting state, with deactivation occurring during task performance^22^. Dynamic interactions among DMN, ECN and SN play an important role in the shift between resting and focused attention. The ECN and/or SN may negatively regulate activity in the DMN. The ECN and SN exert control over the DMN^40^ and may play a preferential role in trial-to-trial adaptive control; such adaptation may involve balancing internally-oriented DMN activity and externally-oriented task-related ECN/SN activity. The DMN global network topology is dynamically linked to brain state and it dramatically changes over the 24-hour day during different brain states^45^. (**B**) Stress increases connectivity of DMN, shifting towards an alerted default mode^44^. Acute stress responses upregulate the SN. Altered connectivity within the SN is paralleled by changes in the ECN. Prolonged stress leads to DMN hyperactivity^47^; the DMN is intrinsically related to both SN and ECN. Microgravity may also induce changes in resting-state functional connectivity of the DMN.

Being extremely complex, adaptation to a constantly changing environment occurs flexibly through several mechanisms, including physiological, behavioral, cognitive, and environmental demands^21^. The default mode network (DMN)^22–25^ and gut feeling^26^ may serve a broad adaptive process. The DMN consists of large-scale brain dynamics^27–29^, comprising interacting subsystems linked by hubs (see Fig. 1), such as the anterior medial prefrontal cortex (anterior mPFC) and posterior cingulate cortex (PCC)^30^. The dorsal medial subsystem includes the temporoparietal junction (TPJ), important for adaptation in nature^31–34^.

DMN activity is also driven by highly-coupled areas of the parietal cortex (see Fig. 1), linked to the medial orbitofrontal cortex (mOFC)^35–38^, which shares reciprocal connections with the basolateral amygdala, the anterior cingulate cortex (ACC), the hippocampus, and the posterior para hippocampal cortex. The lateral orbitofrontal cortex (lOFC) coactivates with cognitive control regions of the salience network (SN), including the bilateral anterior insula cortex (AIC) and rostral ACC, which project to the dorsomedial and ventroanterior nuclei of the thalamus. The mOFC may be necessary for encoding subjective stimuli and for reward-guided learning^36^, while the lOFC may be crucial for reversal learning^28,29^.

Of the major brain networks, the DMN, the executive control network (ECN), and the SN may be involved in the adaptation to a novel environment^39–42^ (see Fig. 1). While the DMN’s activity is increased in response to internally focused cognitive processes, the ECN (distributed in the frontoparietal areas) engaged in externally directed tasks, is implicated in the management of exogenous cognitive functions. The SN, anchored primarily in the bilateral AIC and dorsal ACC, allocates resources to the most salient events among internal (DMN) and external (ECN) activities^43^.

The DMN’s organization may vary with the time of day between the usual default mode and an alerted default mode across different brain states during resting and task states (see Fig. 1)^30,44,45^. Lin *et al*.^30^ showed that static and dynamic DMN nodal topology is associated with upcoming cognitive task performance, and suggested that the core node PCC within the DMN plays a key role in supporting cognitive function. In response to stress, the DMN exhibits increased resting-state functional connectivity to key nodes of the SN (AIC, dorsal ACC [dACC] and amygdala), sensorimotor regions and higher-order visual areas, indicative of a shift to an alerted default mode^44^. Exposure to prolonged stress leads to functional changes in several large-scale brain networks^30,46,47^.

Multimodal integration of internal and external sensory signals occurs in the AIC through salience processing and interceptive predictions^48^. The AIC is responsible for body awareness and gut feeling^26,49^. In particular, the rostral-frontal insular cortex^50^ plays a major role in the integration of visceral sensory information arising from baroreceptors and chemoreceptors within the cardiovascular system, and it is involved in the efferent control of cardiovascular functions, behaviors usually processed by the unconscious mind.

The DMN plays an adaptive role in internal mentation^24^. Subliminal stimuli can alter behavior via non-conscious processes^51–59^. Behavior adjustment is flexible and occurs on a trial-to-trial bias. Neural models of behavior elicited by non-conscious stimuli implicate the prefrontal and cingulate cortices in regulation of subcortical brain regions linked to impulsive and largely non-conscious stimulus perception over time.

Non-consciously processed stimuli activate the visual cortex, insula (a brain area involved in eventual interceptive awareness) and ACC^51^, forming a basis for conscious perception. The insular cortex, receiving sensory inputs from both interoceptive and exteroceptive sources, is thought to integrate these multimodal signals. fMRI studies showed that internal bodily signals processing in the insular cortex modulates exteroceptive awareness. The AIC has been implicated in temporal processing, which may relate to its role in integrating internal interoceptive temporal cues with external signals. Consciously and unconsciously acquired relational memories may be harbored within a single, cohesive hippocampal-neocortical memory space, where they interact with each other^52,53^.

**Figure 2:**
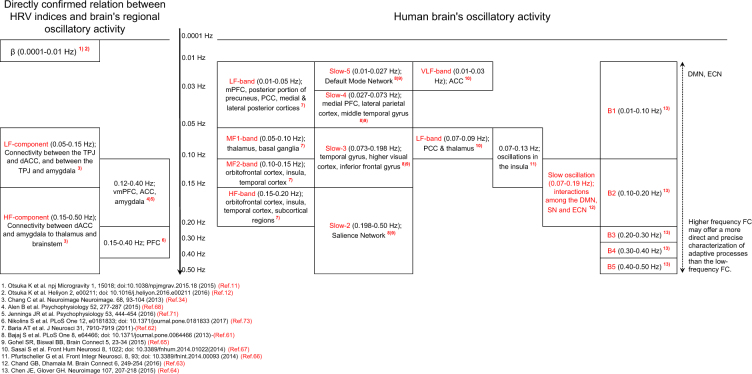
Relationship between HRV indices in different frequency ranges and brain’s regional oscillatory activity. BOLD signals measure brain oscillatory activity by studying changes in spectral power within various frequency bands during resting-state and stimulus-evoked activity. Resting-state fMRI studies showed BOLD fluctuations indicative of functional integration between brain regions occurring over multiple frequency bands, from slow-5 (0.01–0.027 Hz) to slow-1 (0.50–0.75 Hz). Dynamic functional connectivity patterns are tightly linked to adaptive behavior^25^. Resting-state networks consist of frequency-specific subcomponents, shifts in power of brain oscillations during a task occur in specific frequency bands in brain-specific regions. Frequencies in the 0.10–0.20 Hz range play a significant role for functional connectivity^61^. Oscillation in the 0.07–0.19 Hz range mediate the interaction within and between the DMN, SN and ECN nodes^63^. Slow-3 frequency band (0.073–0.198 Hz) reflects intrinsic oscillations in the insula^61,63,65,66^. HRV varies in concert with changes in brain functional connectivity; the heart and brain are connected bi-directionally. HRV may serve as a proxy for ‘vertical integration’ of the brain system^21^ in which DMN plays an important role in adaption to microgravity. HRV activity is associated with structures and functions of brain’s DMN, SN and ECN neural networks^34^.

The human brain oscillates in harmony with frequency-specific subcomponents of several brain regions^60–67^. Brain oscillatory activity is usually measured by blood oxygen level-dependent (BOLD) signal, in terms of power within various frequency bands. BOLD oscillatory activity has been examined in the resting-state and during stimulus-evoked activity. The frequency-dependent power differs in different brain regions. The DMN is also composed of distinct frequency-dependent regions (see Fig. 2). According to resting-state fMRI studies, functional integration among brain regions occurs over multiple frequency bands ranging from slow-5 (0.01–0.027 Hz) to slow-1 (0.50–0.75 Hz). Dynamic functional connectivity patterns may be tightly linked to adaptive behavior^25,45^.

Resting-state networks may consist of frequency-specific subcomponents. Shifts in brain oscillations’ power during a task occur in specific frequency bands and in a spatially-specific manner. Frequencies in the range of 0.10–0.20 Hz may be important for functional connectivity^61,63,65,66^, mediating interactions within and among the DMN, SN and ECN nodes. The 0.10–0.20 Hz frequency band may reflect intrinsic oscillations in the insula.

The intimate brain-heart connection enunciated by Claude Bernard can be studied by analyzing HRV^21^, which may reflect the activity of the coordinating system (see Fig. 2), notably brain functional connectivity, including the DMN^68–74^, which integrates the brainstem nuclei that directly regulate the heart. HRV may provide information on how the brain coordinates with the periphery, and thus may serve to obtain information about the extent of adaptive adjustment.

The circadian system, allowing the measurement of time and the anticipation of environmental changes between light and dark, offered a survival advantage throughout evolution^12,17,18^. The circadian system drives numerous physiological and behavioral processes^17–19^; similar observations have been made in space^10–12,16^. The suprachiasmatic nuclei (SCN) coordinate circadian physiology and behavior by using neuronal and humoral signals that synchronize local clocks within the cells of most organs and tissues. Some of the SCN output pathways serve as input pathways for peripheral tissues^15^. The circadian gene network is present in most living organisms, from eubacteria to humans, and most cells and tissues express autonomous clocks. Disruption of clock genes results in physiological and metabolic dysregulation at neural, molecular, and cellular levels^14,15,18–20^; dysregulation has been verified in astronauts in space^4,10–12,16^.

Briefly, neuroimaging^22–25,30–32^ showed that dynamic functional connectivity patterns are tightly linked to adaptive behavior during rest/sleep^22,25,30,35,75^, and while awake. Adaptation processes are carried out mainly by the DMN^23,24,29–32,44–47^ and operate regardless of consciousness^51–59^. Adaptive responses of the DMN represent a change towards a more salient brain function (see Fig. 1). The DMN selectively shows an elevated connectivity with hubs of the SN, higher-order visual areas that co-vary with new environments^24,44–50^, reflecting an alert default state of the brain^44^, while SN and ECN connectivity remain stable. Adaptation-related changes are associated with shifts in the frequency of brain region-specific BOLD signals (see Fig. 2)^60–67^, which may be reflected in HRV changes mediated by cortico-subcortical pathways regulating the parasympathetic and sympathetic branches of the autonomic nervous system^68–74^.

Understanding the interplay between circadian changes in dynamic fluctuations of brain activity remains a major challenge^7,8^, notably in relation to the resting-state functional connectivity, including the DMN. This question is investigated herein, using HRV endpoints derived from repeated 24-hour ECG records provided by 8 astronauts. We explore (1) ways by which the brain adapts to microgravity; (2) whether DMN space adaptation occurs preferentially at certain circadian stages; and (3) whether there is a common or diverse modes of adaption. We posit that: HRV covaries with diverse fluctuations of brain regions and/or with temporal changes in whole-brain functional connectivity, reflecting adaptation behaviors to microgravity in space.

## Methods

### Subjects

Eight healthy astronauts (6 men, 2 women) participated in this study. Their mean (±SD) age was 51.8 ± 3.7 years. Their mean stay in space was 174.5 ± 13.8 days. Astronauts had passed class III physical examinations from the National Aeronautics and Space Administration (NASA). The study was approved by the NASA and the Japan Aerospace Exploration Agency (JAXA) Institutional Review Boards. Informed consent was obtained from all subjects. A detailed explanation of the study protocol was given to the subjects before they gave written, informed consent, according to the Declaration of Helsinki Principles. All methods were performed in accordance with the JAXA/NASA guidelines and regulations.

### Experimental protocol

Ambulatory around-the-clock 24-hour electrocardiographic (ECG) records were obtained by using a two-channel Holter recorder (FM-180; Fukuda Denshi). Measurements were made five times: once before flight (Control); three times during flight on the International Space Station (ISS): ISS01, ISS02, and ISS03; and once after return to Earth (After flight). The control session was conducted on days 226.7 ± 158.7 (63 to 469) before launch in all but one astronaut who had technical problems with his before-flight record. In his case, a replacement control record was obtained 3.5 years after return to Earth. The three sessions in space were performed on days 21.0 ± 3.2 (18 to 28, ISS01), 73.4 ± 3.8 (68 to 78, ISS02) and 156.08 ± 16.6 (139 to 188, ISS03) after launch, the latter corresponding to 18.1 ± 3.4 days (11 to 21) before return to Earth. The last measurement session was performed on days 70.3 ± 28.6 (37 to 106 days) after return to Earth (After flight).

### Analysis of HRV and measurement of 1/f fluctuations in HR dynamics

Data collection and measurement procedures were conducted as previously reported^10–12^. Briefly, for HRV measurements, QRS waveforms were read from continuous ECG records. The RR intervals between normal QRS waveforms were extracted as normal-to-normal (NN) intervals. The measured NN intervals were A/D converted (125-Hz) with 8-ms time resolution. After the authors confirmed that all artifacts were actually removed and that the data excluded supraventricular or ventricular arrhythmia, time-domain HRV indices, including SDNN, SDANN and Triangular Index (TI), and the conventional frequency-domain measures^76^, including the high frequency (HF) (0.15–0.40 Hz), low frequency (LF) (0.04–0.15 Hz), and very low frequency (VLF) (0.003–0.04 Hz) components, were obtained with the MemCalc/CHIRAM (Suwa Trust GMS, Tokyo, Japan) software^77^. Time series of NN intervals were also processed consecutively in 180-min intervals, progressively displaced by 5 min, to estimate the ultra-low frequency (ULF) component (0.0001–0.003 Hz), and to evaluate the 1/f ^β^-type scaling in HRV, by plotting the log_10_[power] (ordinate) against log_10_[frequency] (abscissa) and fitting a regression line to estimate the slope β. Focus was placed on the frequency range of 0.0001–0.01 Hz (periods of 2.8 hours to 1.6 minutes).

In addition, time series of NN intervals covering 5-min intervals were processed consecutively, and three types of spectral power in eight frequency regions were computed using the Maximum Entropy Method (MEM): 1. LF-band (0.01–0.05 Hz) according to Baria *et al*.^62^; 2. LF-component (0.05–0.15 Hz) and HF-component (0.15–0.50 Hz) according to Chang *et al*.^34^; and 3. B1- (0.01–0.10 Hz; 100 sec to 10 sec), B2- (0.10–0.20 Hz; 10 sec to 5 sec), B3- (0.20–0.30 Hz; 5 sec to 3.3 sec), B4- (0.30–0.40 Hz; 3.3 sec to 2.5 sec) and B5- (0.40–0.50 Hz; 2.5 sec to 2.0 sec) bands according to Chen and Glover^64^. Herein, we call Baria *et al*.’ LF-band “HRV very slow-frequency oscillation (HRV-VSFO)”, Chang *et al*.’s LF- and HF-components “HRV LF- and HF-components”, and Chen and Clover’s B2-band “HRV slow-frequency oscillation (HRV-SFO)”.

### Circadian stage-dependent HRV response to microgravity

Circadian stage-dependent HRV responses to the adaptive processes were assessed by subdividing the 24-hour day into 5 time spans of 3 hours each: late sleep (around 04:30); morning (around 07:30); afternoon (around 14:30); evening (around 21:30); and early sleep (around 00:30).

### Fit of single 24-hour cosine model

Single 24-hour cosine curves were fitted to various HRV endpoints by cosinor^11,12,18,19^ to assess their time structure, especially focusing on the circadian amplitude. The 24-hour cosine model was fitted to 24-hour records of NN intervals, total power (TF), and power in the conventional ULF, VLF, LF, and HF regions of the MEM spectrum.

### Statistical analyses

Estimates of HRV endpoints averaged over 24 hours, or over 3-hour spans to assess circadian stage-dependent effects, were expressed as mean ± SE (standard error). Changes in each HRV index during ISS01, ISS02 and ISS03 were compared to pre-flight (control) by the two-tailed Student t-test.

Estimates of HRV-SFO, HRV LF- and HF-components, and HRV-VSFO during ISS01, ISS02 and ISS03 were compared with those before flight. When they were increased in space, we defined them as a positive response. Any improvement in β was assessed by comparing slopes among the 3 sessions in space (ISS01, ISS02 and ISS03).

We hypothesize that a positive HRV response in two specific frequency regions is indicative of how different astronauts adapt to the space environment. One frequency region is the B2-band (0.10–0.20 Hz; “HRV-SFO”), reflecting the intrinsic oscillations in the insula, involving interactions within and among the DMN, SN and ECN nodes^61,63,65,66^. The other is the LF-band (0.01–0.05 Hz; “HRV-VSFO”), reflecting an activation of the DMN, including the mPFC, posterior parietal cortex, posterior portion of precuneus and PCC.

We propose that a positive HRV-SFO response defines astronauts in Group A (n = 4). In the absence of such a positive response, only some astronauts in Group B may show a positive response in HRV-VSFO (B1, n = 2), while others do not (B2, n = 2). The latter may differ from all other astronauts in their 24-hour HRV endpoints (β, TF, SDNN, SDANN and TI, and circadian amplitude of NN-interval, TF-, ULF-, VLF-, LF-, and HF- components), as tested by the two-tailed Student t-test. As data will accumulate in the future, it will be possible to test the validity of our hypothesis based on the relatively small sample of 8 astronauts thus far.

P-values less than 0.05, adjusted for multiple testing according to Sidak’s correction, were considered to indicate statistical significance. The Stat Flex (Ver. 6) software (Artec Co., Ltd., Osaka, Japan) was used.

## Results

### Characteristics of Group A (increased HRV activity in 0.10–0.20 Hz B2-band)

On the average, astronauts in Group A increased their B2 power by 36.9 ± 8.4% during ISS03 as compared to pre-flight (paired t = 4.374, p = 0.022), whereas astronauts in Group B decreased their B2 power by 22.5 ± 7.5% (paired t = 2.993, p = 0.058). An increase in the HRV-VSFO power was also observed in all 4 astronauts of Group A (see Table 1), suggesting an activation of the DMN. In 2 of them, HRV-VSFO activation occurred during ISS03, in another it occurred during both ISS02 and ISS03, and in another one it occurred only during ISS01. Increase in HRV-VSFO power may be associated with activation in the mPFC, the PCC, the precuneus, and the lateral and medial parietal cortex, approximating brain regions comprising the DMN^62^. In these 4 astronauts of Group A, the intrinsic cardiovascular autonomic regulatory system β improved (see Table 2), confirmed by the fit of a quadratic regression of β as a function of time (x) (R^2^ = 0.568, p = 0.004; t[x] = −3.213, p = 0.007 t[x^2^] = 2.337, p = 0.036). The 3 astronauts showing a positive HRV-VSFO response during ISS03 also improved β during ISS03, and the astronaut with a positive HRV-VSFO response during ISS01 improved β during ISS01 (see Table 2). Adaptation to space in these 4 astronauts may have been mediated via the DMN, shifting to the alerted default mode.

**Table 1:** Classification of subjects into Class A or B based on “slow oscillation”.

	Frequency range (Hz)	Pre		ISS01		ISS02		ISS03		ISS01 vs. Pre		ISS02 vs. Pre		ISS03 vs. Pre	
		Mean	SE	Mean	SE	Mean	SE	Mean	SE	t	p	t	p	t	p
Group A: B2-band response (+)															
LF-band response (+)															
	Case 1														
B2	0.10–0.20	149.8	7.07	138.7	4.70	160.8	4.86	191.6	7.33					4.10	0.0005
LF-b	0.01–0.05	1007.5	54.7	749.0	38.2	952.9	47.9	1235.2	57.1					2.88	0.0408
LF-c	0.05–0.15	459.4	18.8	449.4	14.6	543.9	16.8	664.5	20.5			3.35	0.0085	7.37	<0.0001
HF-c	0.15–0.50	135.9	6.9	102.1	3.4	109.1	3.0	138.3	3.8						
	Case 2														
B2	0.10–0.20	216.7	8.75	346.3	9.00	273.2	8.41	341.9	10.33	10.32	<0.0001	4.65	<0.0001	9.25	<0.0001
LF-b	0.01–0.05	1119.3	52.1	1370.5	53.6	797.7	33.8	1035.5	41.1	3.36	0.0084				
LF-c	0.05–0.15	641.9	22.2	1035.1	26.9	761.0	21.8	837.0	20.7	11.27	<0.0001	3.83	0.0014	6.43	<0.0001
HF-c	0.15–0.50	177.1	6.9	242.3	6.3	170.9	6.2	242.8	10.0	6.95	<0.0001			5.41	<0.0001
	Case 3														
B2	0.10–0.20	25.5	0.90	22.1	0.80	33.6	1.28	30.4	1.04			5.20	<0.0001	3.60	0.0036
LF-b	0.01–0.05	392.9	20.0	443.4	22.5	488.8	24.9	526.6	34.8			3.00	0.0277	3.34	0.0092
LF-c	0.05–0.15	120.0	3.8	106.9	3.3	161.4	5.0	146.4	6.3			6.59	<0.0001	3.60	0.0037
HF-c	0.15–0.50	42.8	1.0	24.0	0.6	34.1	1.0	35.2	1.1						
	Case 4														
B2	0.10–0.20	121.0	5.12	147.2	5.77	150.0	5.43	172.7	6.28	3.39	0.0076	3.88	0.0011	6.37	<0.0001
LF-b	0.01–0.05	880.7	30.8	812.7	36.2	915.3	43.4	1143.9	66.9					3.57	0.0040
LF-c	0.05–0.15	571.0	23.7	538.6	21.0	543.6	19.1	595.8	21.2						
HF-c	0.15–0.50	73.6	2.3	97.9	3.5	89.3	3.8	161.3	5.4	5.79	<0.0001	3.56	0.0041	15.03	<0.0001
Group B: B2-band response (−)															
LF-band response (+)															
	Case 5														
B2	0.10–0.20	169.6	5.98	93.9	3.03	84.5	2.50	96.8	3.59						
LF-b	0.01–0.05	640.8	26.2	1077.1	69.9	1121.6	72.0	937.8	64.6	5.85	<0.0001	6.23	<0.0001	4.26	0.0003
LF-c	0.05–0.15	488.2	15.1	295.4	10.0	280.1	8.6	266.3	9.4						
HF-c	0.15–0.50	120.5	3.1	94.3	3.2	77.5	2.1	214.4	10.4					8.64	<0.0001
	Case 6														
B2	0.10–0.20	132.1	4.74	103.3	3.61	107.6	2.88	113.0	3.29						
LF-b	0.01–0.05	715.3	40.5	715.4	32.9	787.1	38.0	881.3	39.3					2.94	0.0335
LF-c	0.05–0.15	583.0	20.7	448.5	13.9	407.2	10.6	482.7	16.7						
HF-c	0.15–0.50	59.9	2.0	60.7	1.7	79.6	1.9	75.4	1.8			7.11	<0.0001	5.64	<0.0001
LF-band response (−)															
	Case 7														
B2	0.10–0.20	172.9	7.36	152.9	4.81	107.2	3.90	130.9	4.02						
LF-b	0.01–0.05	2661.7	159.3	2251.2	143.9	1420.1	84.6	2305.5	133.4						
LF-c	0.05–0.15	683.7	22.9	617.2	19.6	467.7	16.6	557.8	19.6						
HF-c	0.15–0.50	174.2	5.0	165.0	3.4	107.9	2.6	128.3	2.8						
	Case 8														
B2	0.10–0.20	355.5	12.29	254.9	8.61	327.6	10.06	325.2	11.96						
LF-b	0.01–0.05	2907.3	188.9	2137.7	129.9	2165.7	131.8	2719.0	194.6						
LF-c	0.05–0.15	1110.4	38.1	878.4	34.2	1025.4	34.7	997.1	38.2						
HF-c	0.15–0.50	213.8	8.2	156.9	4.8	194.1	5.4	213.3	8.0						

B2-band (B2-b), HRV-SFO; LF-band: HRV-VSFO; LF-c: HRV LF-component; HF-c: HRV HF-component.

N between 260 and 287; t: Student t; p: P-value (Sidak correction).

**Table 2:** Changes of the intrinsic cardiovascular regulatory system β during 6-month space flight in each astronaut classified into Group A or B.

Case	Pre		ISS01		ISS02		ISS03		ISS02 vs. ISS01		ISS03 vs. ISS01		ISS03 vs. ISS02	
	Mean	SE	Mean	SE	Mean	SE	Mean	SE	t	p	t	p	t	p
	Group A: B2-band response (+)													
	LF-band response (+)													
1	−1.0584	0.0275	−1.0564	0.0213	−0.8186	0.0150	−0.9728	0.0140	9.14	<0.0001	3.28	0.0111	−7.53	<0.0001
2	−1.1645	0.0199	−0.9698	0.0123	−1.1248	0.0171	−0.9050	0.0075	−7.34	<0.0001	4.48	0.0001	11.73	<0.0001
3	−1.3017	0.0205	−0.8700	0.0144	−0.7900	0.0267	−0.8832	0.0173	2.64	0.0843	−0.59	0.9997	−2.93	0.0356
4	−1.1761	0.0157	−1.0009	0.0121	−0.9159	0.0139	−1.0134	0.0191	4.61	0.0001	−0.55	0.9998	−4.13	0.0004
	Group B: B2-band response (−)													
	Group B1: LF-band response (+)													
5	−1.0258	0.0140	−0.8483	0.0232	−0.6810	0.0192	−0.7753	0.0292	5.55	<0.0001	1.96	0.4082	−2.70	0.0699
6	−1.2171	0.0140	−1.1413	0.0209	−1.0638	0.0116	−1.0722	0.0182	3.25	0.0126	2.50	0.1212	−0.39	1.0000
	Group B2: LF-band response (−)													
7	−1.0698	0.0267	−0.8535	0.0242	−0.9988	0.0260	−0.9741	0.0258	−4.09	0.0005	−3.41	0.0071	0.68	0.9990
8	−0.9864	0.0270	−1.0370	0.0228	−0.8830	0.0147	−0.8484	0.0249	5.67	<0.0001	5.58	<0.0001	1.19	0.9303

B2-band: HRV-SFO; LF-band: HRV-VSFO.

N between 230 and 255; t: Student t; p: P-value (Sidak correction).

In 3 of the 4 astronauts of Group A the power of the HRV LF-component increased (see Table 1), suggesting an activation of the TPJ, which serves a broader adaptive purpose. The TPJ provides widespread connectivity to the dACC, insula, amygdala, thalamus and brainstem, likely playing a role on salience processing and autonomic control during adaptation to space.

The HRV HF-component power also increased in 2 of the 4 astronauts of Group A (see Table 1). Such an increase is accompanied by increases in functional connectivity between the dACC and cingulate cortex, basal ganglia, thalamus, amygdala and the brainstem, independent of connectivity between dACC and TPJ^34^.

These results illustrate the complexity of adaptive responses, even though the DMN played a key role in astronauts of Group A with increased activity of the “HRV-SFO”. The response to long-term exposure to microgravity, achieved diversely in different astronauts, elicits profound changes needed towards a more salient mode of brain function requiring a higher interconnectivity among the DMN, SN, and/or ECN, switching to the alerted default mode (see Fig. 1).

### Circadian challenges of Group A astronauts in adaptation to microgravity in space

When subdividing the 24-hour day into 5 time spans of 3 hours each, centered around 04:30 (late sleep), 07:30 (morning), 14:30 (afternoon), 21:30 (evening) and 00:30 (early sleep), the adaptive behavior involving the DMN (switch to the alerted default mode) was found to be circadian stage-dependent in the Group A but not in the Group B astronauts (see Table 3). Overall, LF power in Group A was increased during ISS3 compared to pre-flight by 159 ms^2^ (41%) (paired t = 4.399, p = 0.022), more so during early sleep (335 ms^2^, paired t = 3.493, p = 0.040) than at other circadian stages. In Group B, however, LF power was decreased overall by 128 ms^2^ (38%) (paired t = 4.133, p = 0.026), particularly in the morning (236 ms^2^, p = 0.054) and afternoon (309 ms^2^, p = 0.004). Adaptive behavior was thus activated preferentially in the evening as well as during early sleep in the 4 astronauts of Group A.

**Table 3:** Circadian variation of activites of the brain’s network, estimated by HRV, in Groups A and B.

Case		Pre		ISS03		t	p
		Mean	SE	Mean	SE		
		Group A					
		Late Sleep (04:30)					
1	LF	914.7	84.3	682.1	39.6		
	HF	363.1	22.1	103.7	6.0		
2	LF	749.6	57.4	732.3	61.1		
	HF	283.3	24.4	276.8	23.5		
3	LF	84.0	7.5	199.1	14.9	6.89	<0.0001
	HF	49.6	2.3	40.7	2.5		
4	LF	318.5	38.7	825.6	83.5	5.51	<0.0001
	HF	63.7	4.3	225.7	14.7	10.60	<0.0001
		Morning (07:30)					
1	LF	404.1	35.6	552.4	53.4		
	HF	103.1	10.8	103.3	8.3		
2	LF	704.8	46.7	873.3	59.5		
	HF	216.1	24.1	156.6	7.0		
3	LF	130.0	9.8	138.6	12.8		
	HF	48.9	2.9	46.9	3.8		
4	LF	596.4	60.1	764.7	46.9		
	HF	74.4	6.3	136.2	9.1	5.6	<0.0001
		Afternoon (14:30)					
1	LF	332.0	33.5	412.2	43.5		
	HF	73.2	6.2	107.6	10.8		
2	LF	710.8	66.5	771.4	51.1		
	HF	151.6	12.0	173.0	7.3		
3	LF	115.3	6.9	124.5	8.2		
	HF	41.7	1.9	31.7	1.8		
4	LF	480.5	47.5	481.4	40.7		
	HF	60.4	4.1	137.3	6.1	10.49	<0.0001
		Evening (21:30)					
1	LF	206.0	21.0	560.6	33.0	9.06	<0.0001
	HF	74.6	4.5	126.8	5.9	7.02	<0.0001
2	LF	481.2	46.2	847.4	63.3	4.67	0.0002
	HF	146.5	8.0	201.9	16.1	3.08	0.0335
3	LF	136.4	11.2	102.1	10.7		
	HF	46.6	3.1	27.6	1.5		
4	LF	345.5	71.9	488.2	60.8		
	HF	61.3	6.5	165.1	11.9	7.64	<0.0001
		Early Sleep (00:30)					
1	LF	471.3	43.0	938.6	65.9	5.94	<0.0001
	HF	190.5	16.8	180.6	8.7		
2	LF	456.4	47.1	987.9	70.0	6.30	<0.0001
	HF	190.8	17.6	558.5	35.4	9.31	<0.0001
3	LF	90.7	9.2	275.7	30.0	5.90	<0.0001
	HF	39.6	3.2	29.8	2.1		
4	LF	642.0	74.5	798.6	66.8		
	HF	74.7	9.8	287.3	18.3	10.25	<0.0001
		Group B1					
		Late Sleep (04:30)					
5	LF	494.0	44.2	426.5	34.7		
	HF	142.8	10.7	469.7	13.4	19.04	<0.0001
6	LF	645.6	63.4	505.2	63.6		
	HF	90.6	8.1	81.9	2.6		
		Morning (07:30)					
5	LF	362.5	25.3	199.3	17.2		
	HF	109.9	5.5	87.3	6.7		
6	LF	574.0	74.6	506.4	33.2		
	HF	59.6	7.0	80.0	4.6		
		Afternoon (14:30)					
5	LF	645.7	35.3	232.5	21.0		
	HF	171.9	9.3	89.4	7.5		
6	LF	489.4	31.8	267.5	36.3		
	HF	45.4	2.8	38.6	4.7		
		Evening (21:30)					
5	LF	558.1	41.4	269.2	21.1		
	HF	98.5	5.0	261.9	17.5	8.96	<0.0001
6	LF	391.8	43.0	459.6	23.1		
	HF	58.5	6.2	67.0	2.5		
		Early Sleep (00:30)					
5	LF	378.0	35.1	258.5	23.3		
	HF	125.2	8.3	426.6	18.5	14.83	<0.0001
6	LF	630.9	61.6	506.5	70.1		
	HF	87.8	7.2	87.2	3.7		
		Group B2					
		Late Sleep (04:30)					
7	LF	666.0	62.6	757.2	68.4		
	HF	244.7	14.3	169.8	8.5		
8	LF	1411.9	72.8	1894.1	91.0	4.14	<0.0001
	HF	428.6	22.3	435.1	20.2		
		Morning (07:30)					
7	LF	865.5	95.1	569.9	41.3		
	HF	184.4	14.6	141.8	6.7		
8	LF	1301.9	80.4	885.0	69.1		
	HF	256.0	16.3	180.2	11.4		
		Afternoon (14:30)					
7	LF	778.8	58.1	494.5	37.9		
	HF	135.7	8.8	109.8	4.6		
8	LF	1090.8	106.1	772.7	61.2		
	HF	150.5	8.6	164.8	13.7		
		Evening (21:30)					
7	LF	410.8	35.2	529.8	70.0		
	HF	118.0	4.6	114.6	5.7		
8	LF	444.5	40.3	518.4	48.8		
	HF	135.5	13.3	100.6	9.0		
		Early Sleep (00:30)					
7	LF	612.1	55.0	646.4	60.7		
	HF	183.9	13.5	159.8	8.1		
8	LF	1696.6	96.2	1184.5	76.1		
	HF	329.7	19.7	264.3	10.2		

LF: HRV LF-component (0.05–0.15 Hz).

HF: HRV HF-component (0.15–0.50 Hz).

N between 34 and 37; t: Student t; p: P-value (Sidak correction).

### Characteristics of Group B astronauts (without increased HRV activity in 0.10–0.20 Hz B2-band)

In astronauts of Group B, β also improved, but sporadically (see Table 2). They tried to adapt to microgravity differently. There was no positive response of the HRV LF-component, suggesting that the TPJ did not participate in their adaptive process (see Table 1).

Two astronauts with a positive HRV-VSFO response (Group B1) responded positively in their HRV HF-component (0.15–0.50 Hz) during ISS03 (see Table 1). They showed statistically significant positive responses in B3 (0.20–0.30 Hz), B4 (0.30–0.40 Hz), and B5 (0.40–0.50 Hz) (see Table 4) either during the daytime (see Table 4, top) or during the night (see Table 4, bottom). Since the HRV HF-component reportedly relates to connectivity between dACC, AIC and amygdala to thalamus and the brainstem, brain oscillatory activity may shift from lower-frequency to higher-frequency bands during adaptation processes. Adaptation to microgravity in Group B1 astronauts may thus be initiated by the SN.

**Table 4:** Improvement of β, probably depending on increased Salience network activity in Group B1 (B2-band response −; LF-band response+). B1: 0.01-0.10 Hz; B2: 0.10-0.20 Hz; B3: 0.20-0.30 Hz; B4: 0.30-0.40 Hz; B5: 0.40-0.50 Hz.

Band	Pre		ISS01		ISS02		ISS03		Post		ISS01 vs. Pre		ISS02 vs. Pre		ISS03 vs. Pre	
	Mean	SE	Mean	SE	Mean	SE	Mean	SE	Mean	SE	t	p	t	p	t	p
	Daytime															
	Case 5															
B1	952.9	32.7	892.4	32.9	844.3	26.7	781.9	33.4	1093.9	38.9						
B2	180.1	6.88	93.3	3.91	79.4	2.89	79.4	3.60	141.1	5.44						
B3	39.7	1.73	29.5	1.46	29.2	1.24	47.4	2.94	32.6	1.73						
B4	21.7	0.90	13.0	0.77	10.3	0.54	25.4	1.55	19.0	0.82						
B5	13.1	0.34	9.1	0.78	7.4	0.51	21.2	1.29	6.1	0.18					6.11	<0.001
	Case 6															
B1	1113.8	47.6	1079.6	39.1	1114.2	45.8	1271.7	48.6	1300.4	50.3						
B2	127.7	4.85	108.7	4.05	106.7	3.25	112.7	3.62	134.2	5.67						
B3	18.0	0.74	21.4	0.86	28.8	0.85	26.2	0.86	36.3	1.63	3.01	0.027	9.62	<0.001	7.28	<0.001
B4	6.9	0.27	7.6	0.25	9.8	0.34	10.1	0.35	12.6	0.48			6.75	<0.001	7.19	<0.001
B5	4.3	0.21	4.4	0.13	5.8	0.19	5.9	0.19	7.3	0.31			5.50	<0.001	5.63	<0.001
	Nighttime															
	Case 5															
B1	1174.0	92.7	2203.4	188.8	2232.1	174.5	2018.7	194.2	1533.9	134.6	4.89	<0.001	5.35	<0.001	3.93	0.002
B2	126.8	9.85	95.2	4.59	93.6	4.55	138.6	6.70	119.7	10.70						
B3	87.9	6.41	50.4	2.32	26.1	1.26	215.2	8.90	100.8	7.52					11.61	<0.001
B4	15.0	0.76	12.1	0.32	9.0	0.22	89.6	2.13	11.1	0.75					32.97	<0.001
B5	11.7	0.57	6.8	0.25	4.2	0.11	65.7	1.31	8.5	0.70					37.85	<0.001
	Case 6															
B1	1551.4	176.6	1092.1	97.5	1117.0	93.0	1324.0	125.3	1665.1	167.1						
B2	151.7	14.11	88.9	7.47	110.6	6.27	114.4	7.78	175.1	12.64						
B3	30.1	3.07	31.3	2.07	45.5	3.41	32.2	1.77	57.1	4.37			3.36	0.011		
B4	9.5	0.65	6.7	0.24	9.4	0.31	10.3	0.43	24.4	0.81						
B5	6.7	0.68	4.6	0.18	5.2	0.24	6.6	0.36	14.3	0.46						

N between 181 and 233 (daytime), between 52 and 104 (nighttime); t: Student t; p: P-value (Sidak correction).

The other two astronauts without a positive HRV-VSFO response (Group B2) did not respond in terms of their HRV LF-component or HRV HF-component, except during late sleep (case 8) (see Tables 1 and 3). As compared to the other 6 astronauts, they had statistically significantly larger spectral power of the TF-component measured before flight (p = 0.0007), during ISS01 (p = 0.0102), ISS02 (p = 0.0010), and ISS03 (p = 0.0023) (see Table 5, top). Before flight, they had a higher SDNN (p = 0.0001), SDANN (p = 0.0001) and Triangular Index (p = 0.0002), suggesting a better prognosis in terms of cardiovascular outcome. These HRV indices were significantly larger also during ISS01, ISS02 and ISS03, except for the Triangular Index during ISS03 (see Table 5, top), suggesting that adaptation processes in Group B2 astronauts were independent and different from the brain’s functional connectivity and/or regional cerebral blood flow.

**Table 5:** Comparison of 24-hour HRV indices and circadian amplitude of 2 subjects in group B2 versus the other 6 subjects.

24-hour HRV		Group A and B1 (n = 6)		Group B2 (n = 2)		Student t-test	
		Mean	SD	Mean	SD	t	p
Pre	HR	75.2	9.4	59.5	11.5	−1.962	0.0974
	β	−1.157	0.102	−1.028	0.059	1.647	0.1507
	TF	4725.0	1282.6	15810.2	4353.5	6.379	0.0007
	SDNN	108.50	15.99	211.85	1.06	8.668	0.0001
	SDANN	92.78	12.53	190.90	7.50	10.152	0.0001
	TI	28.22	2.41	52.84	7.19	8.219	0.0002
ISS01	HR	68.9	7.7	60.8	15.6	−1.040	0.3386
	β	−0.981	0.111	−0.945	0.130	0.384	0.7145
	TF	4744.7	1677.6	9369.6	276.9	3.689	0.0102
	SDNN	133.65	18.07	195.00	19.66	4.096	0.0064
	SDANN	114.58	14.10	148.20	14.99	2.888	0.0278
	TI	32.32	5.07	51.60	4.13	4.791	0.0030
ISS02	HR	68.9	7.7	61.2	5.7	−1.276	0.2490
	β	−0.899	0.170	−0.941	0.082	−0.323	0.7574
	TF	4020.1	1040.7	9736.0	1650.0	6.011	0.0010
	SDNN	118.07	26.42	211.90	60.95	3.316	0.0161
	SDANN	100.87	24.08	186.85	35.85	3.988	0.0072
	TI	25.64	3.88	41.60	9.81	3.656	0.0106
ISS03	HR	68.7	7.9	60.4	8.5	−1.267	0.2519
	β	−0.937	0.105	−0.911	0.089	0.307	0.7694
	TF	5122.1	1364.1	12612.3	3217.9	5.068	0.0023
	SDNN	134.10	26.25	210.90	49.78	2.994	0.0242
	SDANN	113.47	22.09	186.95	44.05	3.331	0.0158
	TI	27.92	5.01	38.24	8.18	2.231	0.0672
Post	HR	72.7	7.0	65.0	16.5	−1.008	0.3524
	β	−1.164	0.170	−1.066	0.100	0.749	0.4819
	TF	5606.2	1412.6	11090.0	1920.2	4.451	0.0043
	SDNN	129.07	34.90	198.15	31.32	2.464	0.0488
	SDANN	107.22	32.46	178.50	37.34	2.620	0.0396
	TI	34.03	7.64	43.54	10.90	1.408	0.2088
**Circadian amplitude**		**Group A and B1 (n = 6)**		**Group B2 (n = 2)**		**Student t-test**	
		**Mean**	**SD**	**Mean**	**SD**	**t**	**p**
Pre	NN	69.1	25.9	200.5	40.8	5.560	0.0014
	TF	1240.8	513.7	7781.2	461.3	15.851	<0.0001
	ULF	1140.8	586.3	5957.1	763.8	9.523	0.0001
	VLF	417.7	239.2	3304.5	1640.5	5.019	0.0024
	LF	119.6	92.6	350.7	265.9	2.057	0.0854
	HF	33.9	36.5	85.9	58.1	1.559	0.1701
ISS01	NN	103.1	37.2	182.7	28.4	2.720	0.0347
	TF	2571.6	990.0	3279.8	1427.6	0.807	0.4507
	ULF	2567.7	1064.1	3030.3	2730.7	0.383	0.7148
	VLF	611.3	374.4	1561.2	127.7	3.365	0.0151
	LF	91.4	77.5	339.4	425.5	1.620	0.1564
	HF	33.2	31.4	47.5	26.9	0.572	0.5881
ISS02	NN	88.7	39.5	179.0	9.4	3.040	0.0227
	TF	2329.4	1892.0	7434.7	3286.6	2.859	0.0288
	ULF	2026.4	2056.6	5318.0	4264.9	1.574	0.1664
	VLF	587.6	262.7	1658.6	844.6	3.123	0.0205
	LF	95.1	53.2	364.7	310.5	2.432	0.0510
	HF	27.6	13.8	53.4	47.8	1.363	0.2219
ISS03	NN	107.8	28.0	190.6	32.9	3.513	0.0126
	TF	3405.3	2320.0	6082.1	3484.3	1.285	0.2462
	ULF	3153.1	2045.1	4921.5	4064.2	0.867	0.4192
	VLF	621.8	405.3	2695.9	2236.2	2.579	0.0418
	LF	134.9	100.1	461.5	418.3	2.065	0.0844
	HF	69.0	66.2	78.9	74.6	0.180	0.8630
Post	NN	88.7	50.5	175.8	91.4	1.798	0.1223
	TF	1752.8	613.5	4717.8	1049.4	5.150	0.0021
	ULF	1525.0	626.5	3277.0	518.5	3.519	0.0125
	VLF	485.8	228.3	2394.0	1567.7	3.472	0.0133
	LF	155.0	75.1	425.7	494.5	1.555	0.1709
	HF	39.4	19.7	41.6	45.8	0.103	0.9215

HR: Heart Rate; NN: NN-interval.

### Circadian characteristics of Group B2 astronauts

As compared to the other 6 astronauts, the two astronauts of Group B2 had a statistically significantly larger circadian amplitude of NN-interval (p = 0.0014), TF-component (p < 0.0001), and conventional ULF (p = 0.0001) and VLF (p = 0.0024) components measured before spaceflight. Several of these indices were also significantly larger during ISS01, ISS02, ISS03, and after return to Earth (see Table 5, bottom); circadian amplitudes of the LF and HF components were not significantly different (see Table 5, bottom).

## Discussion

Neuroimaging studies suggest that HRV varies in concert with changes in brain functional connectivity^34,68–74^. If so, HRV could serve as a proxy for ‘vertical integration’ of the brain system in which the DMN widely interacts with other large-scale brain systems and may play a role in the adaptive process to space’s microgravity. Microgravity induces altered blood volume distribution, which disturbs neural mechanisms involved in dynamic cardiovascular coordination^1–4,6,10–12^. Since urgent action is needed to help the neural cardiovascular coordination adapt to the space environment, adaptive responses were examined, based on 24-hour HRV endpoints.

As expected, coordination of physiological functions was diverse and varied over time, with no common path of accommodation methods for successful adaptation. A thorough and extensive analysis of the data uncovered two HRV frequency bands, the HRV-SFO (0.10–0.20 Hz) and the HRV-VSFO (0.01–0.05 Hz) that we propose may classify astronauts in terms of mechanisms they use to adapt to microgravity in space (see Table 1).

Some limitations need to be noted. By necessity rather than choice, neuroimaging could not be performed in space, preventing the direct investigation of the relationship between the DMN, SN, and the circadian system. Comparisons of HRV endpoints were made on 5-min estimates obtained over 24 hours. As a result, serial correlation may have led to P-values that are too liberal. Comparisons between different sessions provided by each astronaut were made using the Student t test rather than the paired t test because some 5-min estimates are occasionally missing due to artifacts in the data. To identify different kinds of adaptation responses to microgravity, many comparisons were made, between records in space versus pre-flight and among the 3 sessions in space. While P-values were adjusted for multiple testing, results only intend to propose a framework for assessing astronauts’ adaptation process. Data to accumulate in future studies will be critical to determine whether the proposed classification remains valid or whether it needs to be refined.

In astronauts with a positive response in HRV-SFO, the intrinsic cardiovascular autonomic regulatory system, gauged by β, improved (see Table 2). The HRV LF-component power increased by 25 ± 8% (paired t = 3.011, p = 0.057), whereas it decreased by 23 ± 8% in Group B astronauts (paired t = 2.942, p = 0.060). We propose that two types of corticostriatal circuits play key roles in complex adaptive behaviors. One is the DMN circuit, acting via the TPJ networks^31–34^, and the other is the SN circuit acting via the lOFC loop^35–38^, including the PFC, anterior insula and ACC. HF-component (0.20–0.50 Hz) activity increased in Group B1, suggesting SN (dACC, anterior insula and amygdala) involvement in the adaptation process^39–43,68,71,73^. Since changes in brain functional networks were not observed in Group B2 (see Table 1), other adaptation mechanisms may have taken place. Their larger circadian amplitudes of TF, SDNN, SDANN and TI compared to the other 6 astronauts suggest that long-term HRV may be more predictive^19,76,77^ than short-term HRV indices in their case (see Table 5, top).

As anticipated, changes in HRV activities during adaptation to space were found to be circadian stage-dependent, being strongest during early sleep (see Table 3), implying a role of the circadian system, which was also observed in Group B2 astronauts, who had more prominent circadian rhythms of several HRV indices as compared to the other 6 astronauts (see Table 5, bottom). The circadian organization is thus likely to play a role in the adaptation process of all astronauts. Results illustrate how diverse adaptation processes can be, primarily involving the dynamics of large-scale brain networks, initiated by the DMN (Group A), sometimes supported by the SN (Group B1), but always coordinated by the circadian system and occurring irrespective of consciousness. Several questions need to be addressed.

First, why is the 0.10–0.20 Hz HRV frequency band important in adaptation? Bajaj *et al*.^61^ observed that brain activity oscillations dynamically changed along time scales of seconds to minutes. Frequencies in the range of 0.10–0.198 Hz played a significant role for functional connectivity, especially in the DMN. Pfurtscheller *et al*.^66^ observed an increase in the frequency range of 0.07–0.20 Hz in association with activation of the insula. The slow oscillation (0.07–0.19 Hz) reportedly mediates the interactions within and among the DMN, SN and ECN nodes for decision-making tasks, the right AIC of the SN causally controlling the DMN and ECN in combination with the dACC^63^. Social stress responses reportedly upregulate the DMN functional connectivity towards an alerted default mode^44^, the DMN being intrinsically related to both SN and ECN nodes, mediated by increased activity of the right AIC and dACC. Increase in 0.10–0.20 Hz HRV power, reflecting an activation of the insula, suggests that the DMN shifted towards a state of increased vigilance to prepare astronauts to detect salient stimuli and reorient attention for a successful adaptation to the space environment (see Fig. 1).

Second, what role does the circadian system play in the adaption process? Ubiquitous circadian changes have gained in interest^13,15,17–20^, notably in relation to neurobehavioral functions. Circadian changes in brain functional connectivity have been observed^78^, in part using resting-state fMRI^79,80^. Neural activity of the brain fluctuates over the course of 24 hours and circadian stage is important when interpreting functional connectivity data.

Several factors contribute to inter-individual variability^20^, including chronotype, age, sleep disturbance and disordered sleep-wake cycle, medication, and circadian disruption caused by lifestyle choices and/or disease conditions. Inter-individual variation was found in the way different astronauts adapt differently to microgravity. DMN activity of Group A astronauts was higher in the evening and early sleep. Increased sleepiness induces neurochemical consequences for the brain at the higher brain performance. Previous studies assumed *a priori* a link between DMN activity and mind wandering^31,37,38^, now substantiated by a greater cerebral BOLD response in the evening^80^, involving the prefrontal cortex (medial and inferior orbital frontal gyrus, and anterior cingulate). In early NREM sleep, the DMN has no measurable change in functional connectivity^75^, which is important since maintenance of these network connections through ongoing spontaneous activity may be of fundamental importance to the living brain. In Group B1 astronauts, the SN was involved in the adaptation process, as was the circadian system. Differences in the adaptive response between nighttime and daytime may reflect inter-individual differences. The involvement of the circadian system is not surprising since brain’s cortical functions are circadian rhythmic, their phase varying across brain regions^81^. In Group B2 astronauts, circadian organization was important, independently of the brain’s functional connectivity.

Third, what are implications for an unconscious activation of adaptive processes? Recent empirical findings in psychology suggest that complex behavior, including adaptive processes, could take place without conscious attention^54,55^. Human senses can handle more than 11 million bits/s: 10 million are processed by the visual system; ~1 million via the ears; 10,000–100,000 via the skin; much fewer by the olfactory system and remaining sensory channels (gustatory and vestibular). Only a minuscule proportion of sensory data (~50 bits/s) is processed by the conscious mind^56^; remaining data are processed unconsciously; that is ~220,000 (11,000,000/50) times as many as data processed consciously.

Unconscious information processing has been described as flexible, sharing many sophisticated characteristics with its conscious counterpart^55,57,58^. Several brain regions are involved in unconscious processes, including the PFC, insula, ACC, hippocampus, amygdala, PCC, precuneus, and thalamus, hubs or subsystems of the DMN. The insular cortex particularly receives sensory inputs from both interoceptive and exteroceptive sources and integrates these multimodal signals^59^.

Fourth, how well does HRV reflect brain oscillatory activity? Several neuroimaging studies provided intimate connections between HRV endpoints and different brain regions, showing bidirectional relations between the heart and brain. Levels of HRV activity are considered to associate with structures and functions of brain’s neural networks, including the DMN, SN and ECN^34^. Based on this information, we examined how astronauts adapt to microgravity by analyzing HRV changes in specific frequency regions linked to specific brain functional networks in repeated 24-hour ECG records over 6 months in space. These associations are extremely complex and will need to be validated in future studies.

New insights regarding the brain’s functional organization obtained from fMRI signals^60^ provide information regarding the spontaneous fluctuations of the resting-state brain. The spatial distribution of brain’s oscillatory activity was examined as a function of four frequency bands^62^: low-frequency (LF: 0.01–0.05 Hz); medium-frequency-1 (MF1: 0.05–0.10 Hz); medium-frequency-2 (MF2: 0.10–0.15 Hz); and high-frequency (HF: 0.15–0.20 Hz). Individual frequency bands had distinct spatial profiles. Frequency-dependent organizing rules for the best characterized resting-state network were observed, LF oscillations having most significant power in the mPFC, PCC, precuneus, lateral and medial parietal cortex, which together approximate brain regions comprising the DMN, the most prominent intrinsically connected network. The MF1 band was localized to the thalamus and basal ganglia, while the MF2 and HF bands associated with the orbitofrontal, insula, temporal cortex and subcortical regions, suggesting that more complex brain areas are dominated by higher-frequency brain oscillations. The DMN, in general resting-state networks, is thus considered to consist of frequency-dependent sub-regions.

Fluctuations in HRV indices appear in harmony with the behavior of networks in brain regions. The mPFC may be a particularly important part of the neural network^21^. Following adaptive changes in amygdala functional connectivity of patients with excessive worrying symptoms over 1 year^72^ showed that HRV represented progressive functional alterations in connectivity between the amygdala and mPFC.

Brain regions were identified where functional connectivity with the dACC and amygdala (key nodes of the salience network) varied in concert with changes in the HRV HF- and LF-components^34^. Increases in HF-component were accompanied by increases in functional connectivity between the dACC and regions including the basal ganglia, thalamus, midbrain and brainstem, and between the amygdala and regions including the basal ganglia, anterior insula and dorsolateral prefrontal cortex. Increases in LF-component also related positively to changes in connectivity between the temporoparietal junction (TPJ) and both the dACC and amygdala, which provides connections to the insula, and is associated with changes in vigilance states and attentional shifts^34^. The TPJ is a key node of the DMN^31–34^. Increase in LF-component suggests that DMN’s role in adaptation to microgravity and in coordination of coordinating functional connections with other brain networks, including the SN.

The mPFC is another important node in both DMN and SN^21,70^, providing an anatomical site for shifting from DMN to SN activation. mPFC activation is positively related to HF-HRV. Despite the lack of overall association between HF-HRV and DMN, a positive association was reported between HF-HRV and mPFC^71^. Merits of long-term HRV assessment over 24 hours in psychophysiological research have been recognized^82^.

HRV is a biomarker reflecting activities of the brain integration system that regulate adaptive physiological adjustments and autonomic outflow to the periphery. Three brain regions were identified to relate to the HRV HF-component, the ventromedial PFC, ACC and amygdala. Brain regions have also been classified relative to likely indicators of sympathetic and parasympathetic (AIC, hippocampal formation, amygdala and dorsal PCC) control^69^.

Caution, however, needs to be exercised in interpreting neuroimaging studies investigating dynamic associations between brain connectivity and HRV^68^. Factors underlying inter-individual differences in HRV have been reported to include brain morphology^74^. The amount of parasympathetic activity of HRV correlated positively with the cortical thickness of an area within the right anterior midcingulate cortex.

In conclusion, assuming that HRV varies in concert with changes in brain functional connectivity, we analyzed adaptive responses of astronauts to examine how brain functional networks dynamically changed over time in space, using repeated 24-hour HRV. Focusing on 2 HRV frequency bands, the HRV-SFO (0.10–0.20 Hz), reflecting a switch to the alerted default mode, and the HRV-VSFO (0.01–0.05 Hz), showing an activation of the DMN, we found that astronauts adapted to microgravity in diverse ways. We propose that adaptation to microgravity occurs primarily according to two primary processes, one involving the dynamics of large-scale brain networks, initiated by the DMN (Group A) and sometimes supported by the SN (Group B1), and another coordinated by the circadian system (all astronauts, particularly those in Group B2). The adaptation process proceeded even in the absence of consciousness. It may thus not be a dream for humans to live on Mars: together with getting strong cooperation from the circadian system, brain neural networks, the DMN in particular, may facilitate adaptation to the novel space environment.
